# Ocular Color Doppler Ultrasound (OCDUS) in Diagnosis and Monitoring of Ophthalmological, Cerebrovascular and Systemic Diseases: A Narrative Review

**DOI:** 10.3390/jcm15062458

**Published:** 2026-03-23

**Authors:** Massimo Venturini, Silvia Malnati, Noemi Teresa Catania, Andrea Coppola, Chiara Recaldini, Aroa Gnesutta, Marianna Ciani, Silvia Tamietti, Emilio Simonini, Alberta Cappelli, Simone Donati, Filippo Piacentino, Federico Fontana

**Affiliations:** 1 Diagnostic and Interventional Radiology Unit, Department of Medicine and Technological Innovation, Circolo Hospital—ASST dei Sette Laghi, University of Insubria, 21100 Varese, Italy; silvia.malnati@asst-settelaghi.it (S.M.); ntcatania@studenti.uninsubria.it (N.T.C.); andrea.coppola@asst-settelaghi.it (A.C.); chiara.recaldini@asst-settelaghi.it (C.R.); aroa.gnesutta@asst-settelaghi.it (A.G.); mciani@studenti.uninsubria.it (M.C.); stamietti@studenti.uninsubria.it (S.T.); filippo.piacentino@asst-settelaghi.it (F.P.); federico.fontana@uninsubria.it (F.F.); 2 Diagnostic and Interventional Radiology Unit, Carlo Poma Hospital—ASST, 46100 Mantova, Italy; emilio.simonini@asst-mantova.it (E.S.); alberta.cappelli@asst-mantova.it (A.C.); 3 Ophthalmology Unit, Department of Medicine and Surgery, Circolo Hospital—ASST dei SetteLaghi, University of Insubria, 21100 Varese, Italy; simone.donati@uninsubria.it

**Keywords:** ocular color Doppler ultrasound, ophthalmological imaging, central retinal vein occlusion, glaucoma, carotid cavernous sinus fistulas, diabetes

## Abstract

Ocular Color Doppler ultrasound (OCDUS) has been underutilized in the past as a diagnostic technique, although several OCDUS-based studies were performed in the last 30 years for diagnosis or monitoring of some ophthalmological, cerebrovascular or systemic diseases. OCDUS can provide quantitative and reproducible measurements of the blood flow of the main orbital vessels at the retrobulbar level. In this narrative review we aimed to investigate the relevance of OCDUS as an imaging modality in several ophthalmological, cerebrovascular and systemic diseases based on the current literature.

## 1. Introduction

Color Doppler ultrasound (CDUS) is a widely used, non-invasive, low-cost, and real-time imaging technique applied across various medical fields as a first-line examination for diagnosing, characterizing, and monitoring numerous pathological conditions. However, its application in the ophthalmological field remains underrecognized, infrequently utilized and rarely included in routine clinical practice.

Major approaches used to study ocular circulation reported in the literature include: scanning laser ophthalmoscopic angiography with fluorescein and/or indocyanine green dye, optical coherence tomography (OCT) and OCT-Angiography (OCT-A), Computed Tomography Angiography (CTA), Magnetic Resonance Angiography (MRA) and also Ocular Color Doppler ultrasound (OCDUS).

Advancements in ophthalmological imaging have significantly improved disease diagnosis and treatment, particularly with the introduction of scanning laser ophthalmoscopy and OCT. OCT provides non-invasive, high-resolution, 3D images of the retina, while OCT-A adds detailed vascular mapping without dye injections, offering a safe alternative to traditional invasive techniques like fluorescein and indocyanine angiography [1,2,3,4]. Although OCT has limitations such as motion and segmentation artifacts, it remains essential for assessing retinal and choroidal health [5,6,7].

Magnetic Resonance Imaging (MRI) and Computed Tomography (CT) are also valuable for vascular and orbital disease diagnosis, but MRI is generally superior for neuro- ophthalmological conditions [8,9,10].

High-frequency ultrasound provides an accurate representation of the eye’s anatomy and is particularly useful when conditions like cataracts or hemorrhages obstruct traditional imaging techniques, such as ophthalmoscopy. It offers a quick, non-invasive method for visualizing the posterior segment of the eye, aiding in diagnosis and pre-operative planning. Ultrasound is fast, easy to use, and has no reported adverse effects at diagnostic energy levels [11]. Unlike CT [12] and MRI, which lack real-time scanning and have limitations in imaging the vitreous and retina, ultrasound provides dynamic, real-time images, allowing experienced examiners to distinguish between conditions such as retinal detachment, vitreous membrane, tumors, and hemorrhages [13].

OCDUS, unlike fluorescein angiography, which provides mainly qualitative data on the distal branches of ophthalmological vessels, offers valuable quantitative information on the blood flow of vessels at the retrobulbar level. This quantitative insight into the hemodynamics of the eye is essential for diagnosing and monitoring various conditions, as it allows for a more detailed understanding of ocular circulation.

The blood flow of the ophthalmic artery (OA), central retinal artery (CRA), short/long posterior ciliary arteries (SPCAs/LPCAs), superior ophthalmic vein (SOV), central retinal vein (CRV), and vortex veins can be accurately assessed through different parameters, such as diameter, patency, and flow direction, for all vessels; peak systolic velocity (PSV); end diastolic velocity (EDV); resistive index for arteries (RI); and maximum velocity (maxV) and minimum velocity for veins (minV).

Reductions in blood flow velocities, for example, in the CRA or the CRV, or an increased RI = (PSV − EDV)/PSV can be important parameters for diagnosing or monitoring different ophthalmological, cerebrovascular or systemic diseases. The RI reflects a measure of arterial stiffness, atherosclerosis and an early sign of vascular damage [14].

OCDUS has emerged as a promising modality for evaluating orbital blood flow. Like other imaging techniques, it supports diagnosis, prognosis, monitoring, and follow-up of a wide range of ophthalmological diseases. Additionally, it has been shown to be an effective research tool, helping to clarify the pathogenesis of several eye conditions and demonstrating potential in assessing treatment responses. The eye’s cystic structure and superficial position serve as a natural acoustic window, making OCDUS the ideal modality of examination. It enables the precise visualization of even small vessels in the retrobulbar area and, as previously said, it allows sampling of blood flow parameters with quantitative data.

Despite these clear advantages, OCDUS is not yet widely adopted in clinical practice. Barriers include the need for highly skilled sonographers, limited availability of advanced OCDUS technology in ophthalmology departments, and the lack of standardized protocols for specific pathologies, which reduces reproducibility. As the technique becomes more refined and standardized, its application in ophthalmology is likely to expand, offering a powerful tool for improving patient care and advancing ophthalmological research.

OCDUS has been used in the past as a complementary imaging technique in diagnosis or monitoring of several ophthalmological diseases, including central retinal vein occlusion (CRVO), central retinal artery occlusion (CRAO), ischemic optic neuropathy, orbital varix, glaucoma, age-related macular degeneration, retinitis pigmentosa, myopia, and tumors; in different cerebrovascular diseases, such as carotid cavernous/dural fistulas and carotid stenosis, as an alternative to transcranial Doppler during cardiac surgery; and in various systemic diseases including diabetes, hypertension, Takayasu arteritis, Graves’ disease, and pseudoexfoliation syndrome.

This narrative review aims to analyze the main applications and the clinical utility of OCDUS in the diagnosis and monitoring of ophthalmological, cerebrovascular and systemic diseases.

## 2. Ocular Color Doppler Ultrasound (OCDUS)

### 2.1. Examination Technique

The ideal patient position for the OCDUS examination is supine with uncrossed legs to avoid influence on venous return. The patient is then instructed to keep the eyelids closed, to look straight, and to avoid ocular movements. The ideal position for the examiner is near the patient’s head, with the probe cable resting on the shoulders behind the neck to minimize its weight. The examiner should gently place his elbow on the patient’s sternum and apply only minimal pressure to the closed upper eyelids with the probe, aided by the use of a generous amount of sterile gel. Comfort and stability of the examiner’s hand holding the probe are crucial to obtain reproducible measurements. In different studies, various positional set-ups have been used, eventually contributing to inconsistencies and variability in the results reported.

A linear, high-frequency (5–15 MHz) probe is preferred to obtain high resolution.

**Figure 1:**
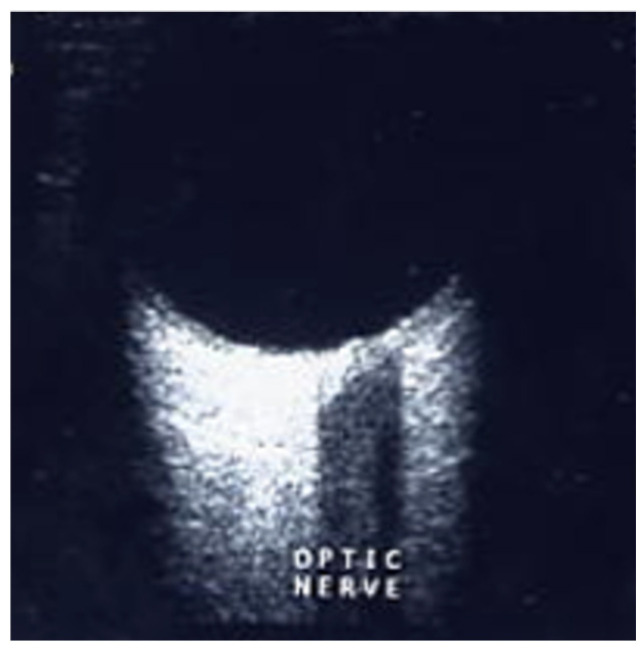
Posterior to the ocular globe, the optic nerve is easily detectable on ultrasound as a tubular hypoechoic structure.

The first step in OCDUS evaluation consists of the visualization of the posterior globe and retrobulbar structures. Optic nerve identification using the B-scan mode can be used as a landmark. The optic nerve can be easily identified as a tubular hypoechoic structure posterior to the eyeball (Figure 1).

Color Doppler is then applied to visualize the main retrobulbar orbital vessels. As previously described, blood flow in the OA, CRA, SPCAs/LPCAs, SOV, CRV, and vortex veins can be accurately assessed through different parameters, including PSV, EDV, RI for arteries and maxV, minV for veins. Almost all orbital vessels, except LPCAs and vortex veins, run parallel to the ultrasonographic beam, facilitating their Doppler spectrum recording. An ultrasound setting with low pulse repetition frequency (PRF) and no wall filter application is recommended to better visualize and record the Doppler waveforms of all orbital vessels except the OA (higher PRF). Careful attention should be paid to angle correction. The direction of the sampling rate has to be in perfect alignment with the angle of the measured vessel to obtain the right correction for the calculated flow velocity [15,16,17,18,19]. The blood flow parameters of orbital vessels in affected eyes from different diseases can be compared with those of normal subjects and, in case of unilateral ocular involvement, also with unaffected eyes. Highly experienced sonographers using Color Doppler (radiologists, ophthalmologists, other specialists), modern, high-level ultrasound technologies, appropriate equipment settings and cooperative patients are essential requirements to perform reliable OCDUS examinations. The routine use of ultrasound, color Doppler and CEUS across multiple clinical fields likely allows the availability of more expensive and advanced ultrasound systems in radiology departments than in ophthalmology units.

### 2.2. Orbital Vessels in Normal Subjects

**Figure 2:**
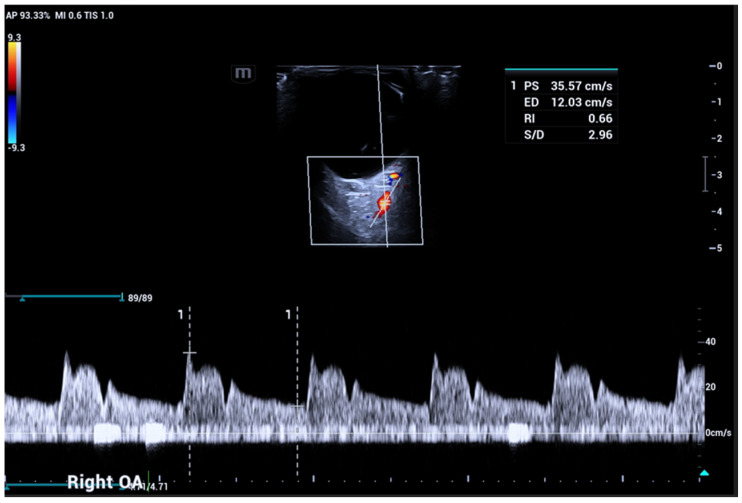
Ophthalmic artery (OA) courses in the superomedial part of the orbit and is usually depicted in red, with a Doppler signal characterized by a peak systolic velocity (PSV) variable from 30 to 60 cm/s: angle correction is essential to obtain reliable velocity measurements.

OA and SOV course through the superomedial part of the orbit and are depicted in red and blue at OCDUS in normal conditions due to the flow direction towards and away from the eye, respectively. The OA usually has a more curvilinear course than the SOV and its blood flow can be easily recorded. The SOV may be occasionally difficult to identify; in this case, a Valsalva maneuver of the patient may increase vessel diameter. The Doppler spectrum of the OA is characterized by a PSV, ranging from approximately 30 cm/s to 60 cm/s, an EDV and a RI ranging from 0.60 to 0.80 (Figure 2).

**Figure 3:**
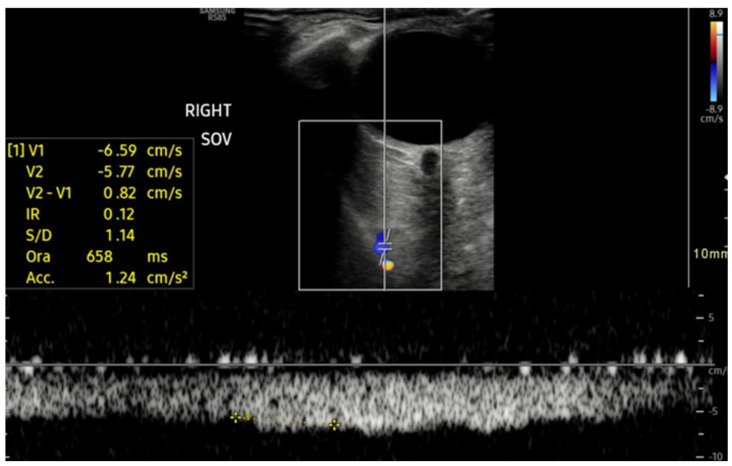
Superior ophthalmic Vein (SOV) courses in the superior part of the orbit and is usually depicted in blue, with a Doppler signal characterized by a continuous venous flow with maximum and minimum velocities (maxV and minV) typically less than 10 cm/s; in some cases, the Valsalva maneuver may be useful to improve visualization.

**Figure 4:**
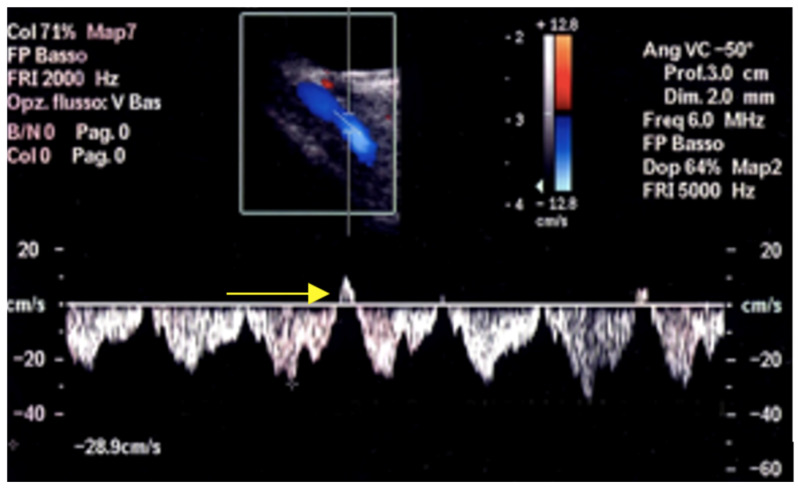
In rare cases, SOV shows a cyclical reversed blood flow (arrow) related to the cardiorespiratory kinetic.

The spectral waveform of the SOV is usually a typical continuous venous flow with minimal differences between maximum and minimum velocities, usually under 10 cm/s (Figure 3), but in rare cases with short, cyclical flow reversal (Figure 4) related to the cardiorespiratory kinetic [20].

**Figure 5:**
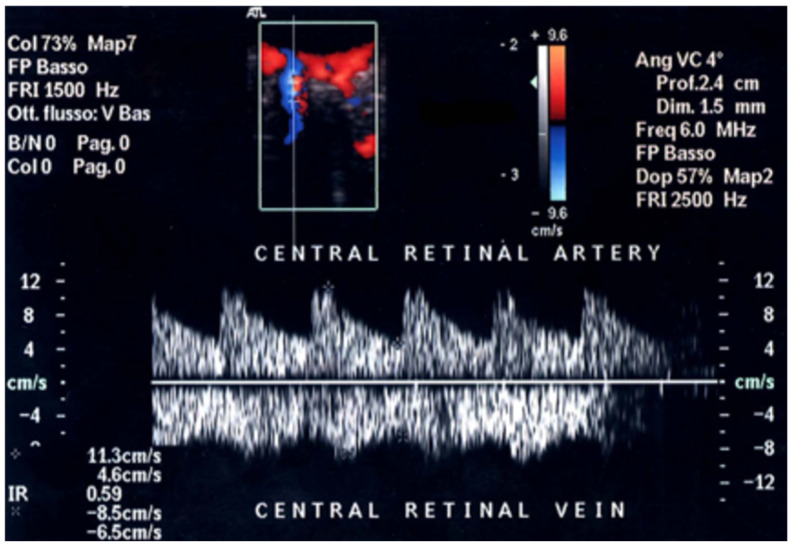
Central retinal artery (CRA) and central retinal vein (CRV), depicted in red and blue respectively, course together within the optic nerve, have a common adventitia and their Doppler signal can be easily distinguished in positive (CRA) and negative (CRV). CRA PSV in normal subjects usually varies from 10 to 16 cm/s, while CRV maxV varies from 5 to 8 cm/s.

The CRA and CRV, which are crucial for visual acuity, course together within the optic nerve, share a common adventitia, allowing simultaneous Doppler recording, with a pulsatile arterial flow in positive and a continuous venous flow in negative [21]. The peculiar course of CRA and CRV into the optic nerve makes their Doppler waveforms easily recordable, particularly at the optic nerve head, despite the low flow velocities (Figure 5).

**Figure 6:**
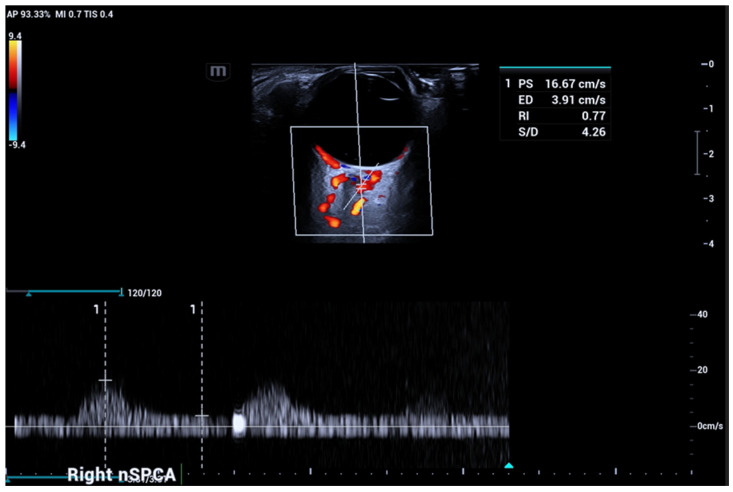
Nasal and temporal short posterior ciliary arteries (SPCAs) course adjacent and parallel to the optic nerve.

In normal subjects, CRA PSV (range 10–16 cm/s) usually corresponds to approximately double the CRV maxV (range 5–8 cm/s). The nasal and temporal SPCAs run strictly adjacent and parallel to both sides of the optic nerve (Figure 6) with low PSV (range 6–18 cm/s).

Their positive Doppler waveform can be distinguished from that of the CRA due to the lack of a negative venous flow, as is typical in the simultaneous Doppler recording of central retinal vessels [20,22].

The SPCAs are responsible for the perfusion of the anterior segment of the optic nerve, its prelaminar part, and the peripapillary part of the choroid.

**Figure 7:**
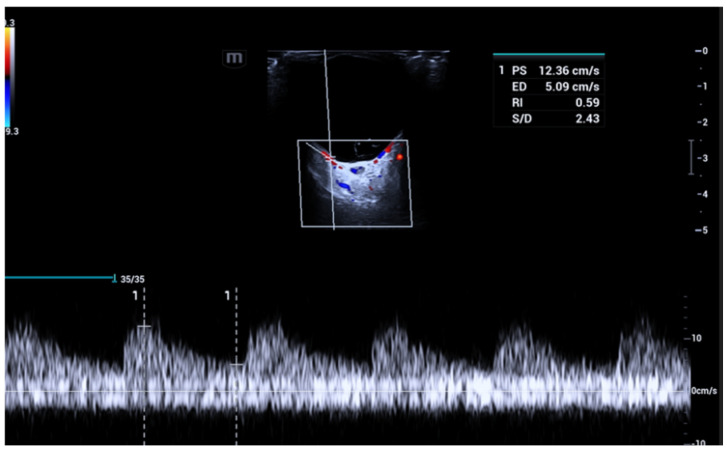
Long posterior ciliary arteries (LPCAs) are depicted in red, coursing parallel to the ocular globe and are often perpendicular to the ultrasound beam; therefore, their Doppler signal may be more difficult to record.

**Figure 8:**
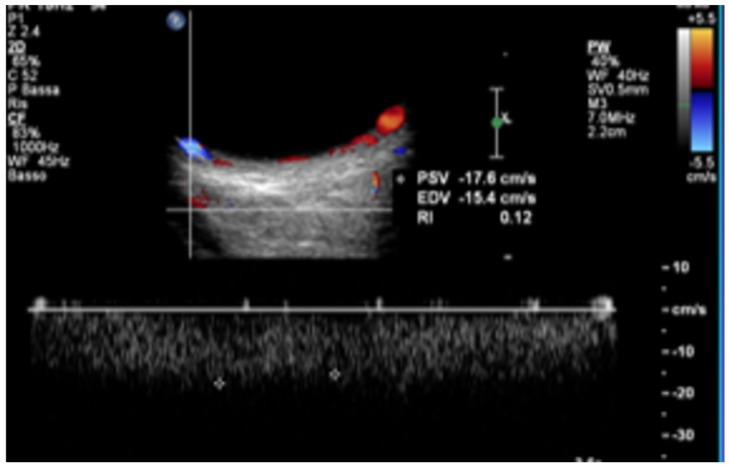
Vortex veins, depicted in blue, course parallel to the ocular globe and are often perpendicular to the ultrasonographic beam. As a result, their Doppler signal may be more difficult to record.

LPCAs (Figure 7) and vortex veins (Figure 8) are located in the posterior part of the ocular globe, and their course, parallel to the eyeball, perpendicular to the ultrasound beam, makes their Doppler spectrum sampleable with difficulty; if necessary, a steering application can be useful in these cases.

In normal conditions, inferior ophthalmic vein (IOV) blood flow is poorly characterizable with OCDUS: it may be recorded in case of its diameter enlargement, for example, in case of high-flow carotid-cavernous fistulas.

## 3. Ophthalmological Diseases

### 3.1. Central Retinal Vein Occlusion (CRVO)

**Figure 9:**
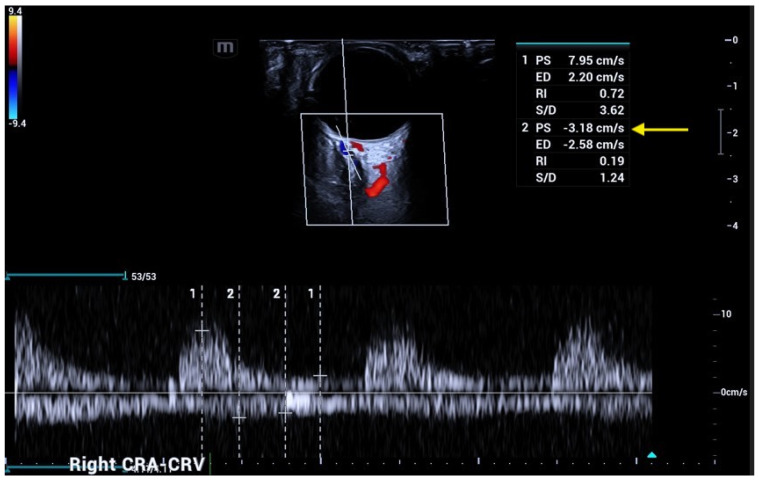
A patient affected by central retinal vein occlusion (CRVO): OCDUS shows a reduced CRV maxV of about 3 cm/s (arrow).

Among orbital vascular disorders, central retinal vein occlusion (CRVO) represents the second most common cause of visual loss after diabetic retinopathy (DR). Prevalence rates reported from population-based studies range from 0.1% to 0.5% of the middle-aged to older age groups [19,20,21]. Arteriosclerosis, hyperviscosity, and coagulation disorders may favor the onset of CRVO [23]. A constriction [24] and an increased thickness [25] of the lamina cribrosa, venous thrombosis site, were demonstrated in CRVO patients. A rapid diagnosis of CRVO, causing a sudden unilateral visual loss in the affected eye, and its differentiation between ischemic and non-ischemic forms for its prognosis is essential [26]. In all cases of CRVO, a marked decrease in CRV velocities (Figure 9) is reported in the literature [27,28,29,30]. These findings were observed in several studies, sometimes revealing significant differences among patients with more severe ischemic, non-ischemic, and branch-type retinal vein occlusion (RVO). However, no significant changes in CRV flow velocities were reported in patients with branch-type RVO compared with those in control subjects and in unaffected eyes [26].

Baxter and Williamson followed 20 patients with CRVO by OCDUS over one year and observed a persistent change in RI and an improvement in venous velocities, with no correlation with prognosis or visual outcome [29]. They also demonstrated that a minV in CRV lower than 3 cm/s at presentation was correlated with the risk of development of neovascularisation [31,32]. Instead, in another study enrolling 102 patients, Arsene et al. [31] found that the minV in the CRV remained lower in ischemic-type eyes than it did in eyes of control subjects and in patients with CRVO one year after hemodilution, whereas the minV in the CRV was similar in both affected and unaffected eyes in patients with the non-ischemic and branch types of RVO. The CRA flow velocities were similar in affected and unaffected eyes in all groups [31]. These results demonstrated the involvement of veins and not arteries in the process of central RVO. Differences in the recanalization of the CRV or in the development of collateral vessels might explain the divergent results between the studies [33]. In our unpublished clinical experience, in almost 60 patients using OCDUS in CRVO, a CRV maxV of less than 5 cm/s was found with a reduction proportional to the thrombosis severity, more evident in the ischemic forms. Usually, a significant difference was found in CRA flow velocities between the acute phase and the follow-up: in the acute phase, CRA PSV were similar to unaffected eyes (more than 10 cm/s), while during the follow-up (after about three months), the behavior of the CRA became similar to the CRV in terms of flow velocities reduction, usually less than 10 cm/s. In our opinion, an outflow reduction is likely to be progressively accompanied by a proportional inflow reduction. This personal observation was also confirmed at emergency OCDUS performed in about 9–10 cases of central retinal artery occlusion (CRAO): in the acute phase, CRA PSV (range 4–8 cm/s) was significantly reduced if compared with normal subjects or unaffected eyes (range 10–14 cm/s) with preserved CRV maxV (more than 5 cm/s), while in the subacute/chronic phase, a proportional reduction in CRV maxV was recorded. In the controversial management of CRVO, a safe and effective therapy has never been demonstrated [34]. Actually, no significant increases in OCDUS of CRV flow velocities during the follow-up were recorded after intravitreal injections of corticosteroids [35,36], radial optic neuropathy [37], panretinal photocoagulation [38] or systemic therapies [39].

### 3.2. Central Retinal Artery Occlusion (CRAO)

**Figure 10:**
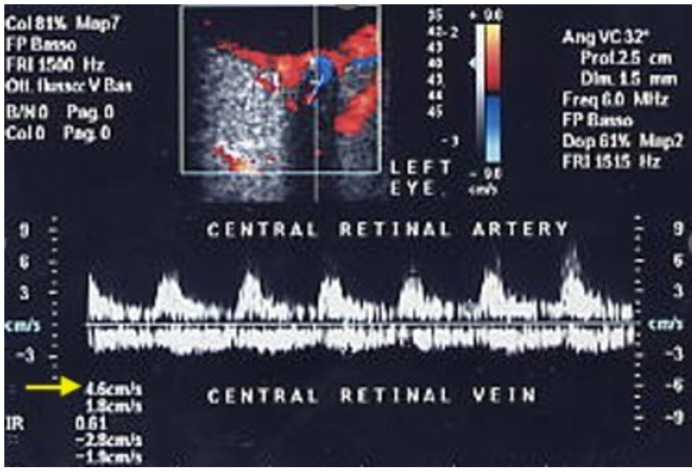
A patient affected by central retinal artery occlusion (CRAO): OCDUS shows a reduced CRA PSV of about 4.6 cm/s (arrow).

Central retinal artery occlusion (CRAO), rarer but more severe than CRVO, is an ophthalmological emergency causing sudden, profound visual loss due to an occlusive thrombus or an embolus. It can also occur as a complication of carotid artery occlusion [40], carotid endarterectomy or stenting [41], or more recently, of cosmetic facial filler injections [42]. The incidence is estimated to be 1 in 100,000 people, accounting for approximately 1 in 10,000 ophthalmological outpatient visits [43]. A prospective study of 260 eyes with CRAO demonstrated that 80% of patients experienced profound monocular visual loss, with a visual acuity of ≤20/400 [44,45]. OCDUS may aid in the diagnosis of CRAO (Figure 10).

In a preliminary experience-based study, Williamson TH et al. [46] reported the absence of arterial color Doppler signals within the optic nerve in three of four patients: this finding may be explained by the limited sensitivity of early-generation ultrasound technology available more than 30 years ago. Using modern ultrasound technologies, OCDUS in patients with CRAO showed a recordable CRA blood flow with a PSV less than 10 cm/s [47]. Moreover, the PSV/EDV ratio was reduced proportionally to the severity of CRAO.

### 3.3. Non-Arteritic Anterior Ischemic Optic Neuropathy (NAION)

**Figure 11:**
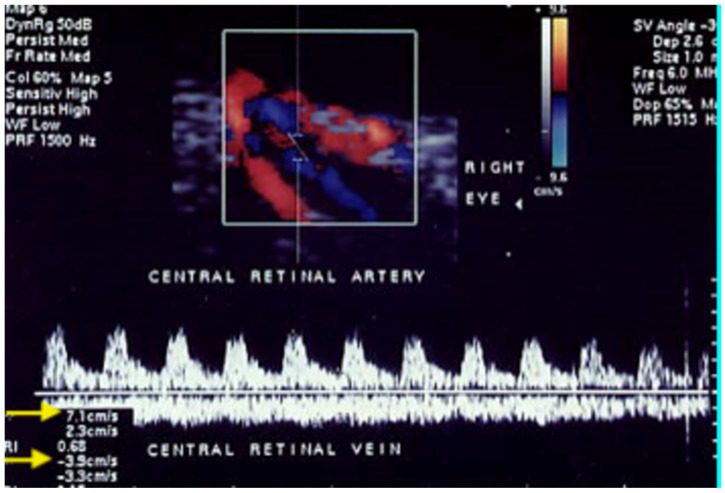
A patient with NAION shows a simultaneous reduction in CRA PSV of about 7 cm/s (arrow) and CRV maxV of about 3.9 cm/s (arrow) at OCDUS.

Anterior ischemic optic neuropathy occurs more frequently than posterior ischemic optic neuropathy. Non-arteritic anterior ischemic optic neuropathy (NAION) is the most common type of anterior ischemic optic neuropathy [48]. NAION is a vision-threatening disorder in elderly individuals, causing unilateral or bilateral vision loss. Although the pathogenesis of NAION remains unclear, several risk factors may lead to regional hypoxemia-related disk edema, likely due to a circulatory insufficiency of the SPCAs resulting in optic nerve laminar infarction [49]. Giant cell arteritis, a systemic vasculopathy that affects large- to medium-sized arteries, is another cause of anterior ischemic optic neuropathy through involvement of optic nerve microcirculation [50,51,52]. Findings regarding arteritic anterior ischemic optic neuropathy (AAION) and NAION are not unequivocal, but both forms can result in permanent visual loss. Nevertheless, a reduction in CRA flow velocities (Figure 11) has been previously reported [53,54].

In 25 patients affected by unilateral NAION compared with controls, Kaup P et al. reported lower PSV and EDV at OCDUS in CRAs and nasal SPCAs, but not in temporal SPCAs. In our opinion, measurements of CRA blood flow are more reproducible and reliable than those of nasal or temporal: CRA blood flow can be easily recorded within the optic nerve (for example at the optic nerve head), whereas temporal and nasal SPCAs are multiple and more challenging to assess consistently at the same location. SPCAs evaluation may be more useful for RI determination in other ophthalmological diseases, such as glaucoma.

### 3.4. Orbital Varix

Orbital varix is a rare, congenital condition encountered in the second or third decade of life, usually determining intermittent, positional exophthalmos [55]. It was previously described as a varix of the vortex vein, which may be confused with other orbital lesions [56], such as choroidal melanoma [57]. Differently from computed tomography or magnetic resonance, OCDUS can characterize in real time the venous blood flow of the orbital varix [58]. A Valsalva maneuver can facilitate its identification and subsequent venous nature demonstration at OCDUS in case of an uncertain diagnosis [59,60,61].

### 3.5. Glaucoma

**Figure 12:**
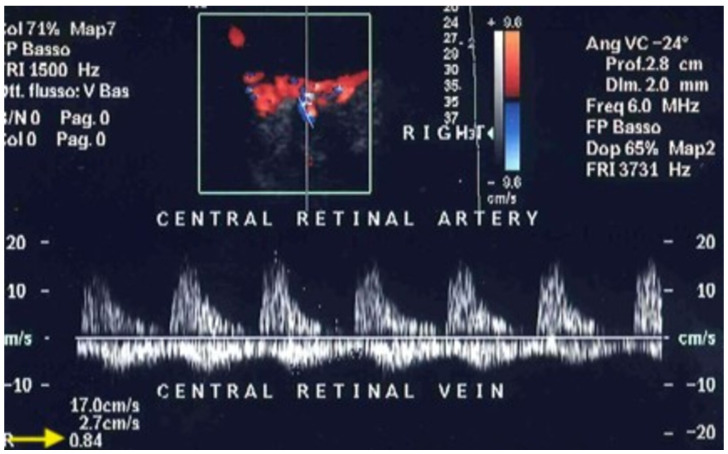
A patient affected by glaucoma shows an increased resistive index (RI) of 0.84 (arrow) in the CRA: usually, in glaucomatous patients, an increased RI is evident at OCDUS in CRAs and SPCAs, not in OAs.

Glaucoma is a neurodegenerative disease caused by the loss of retinal ganglion cells, with characteristic glaucomatous damage of the optic nerve and visual field defects [62]. This condition manifests as a painless and irreversible loss of peripheral vision, potentially resulting in blindness if not promptly treated. The global prevalence of glaucoma in 2014 was 3.54%, and the number of glaucoma patients, aged 40–80 years, is estimated to increase by 73%, from 64.3 million in 2013 to 111.8 million in 2040 [63]. Glaucoma is a multifactorial disease, and optic nerve damage is thought to arise from a complex interplay [56]. Elevated intraocular pressure remains the primary and most studied risk factor for glaucoma’s onset and progression. Other factors include: vascular dysregulation, decreased axoplasmic flow within the retinal ganglion cells’ axons, oxidative stress, and genetic background [64,65]. According to the “vascular theory”, glaucoma results from insufficient intraocular blood flow, with reduced blood flow now recognized as an established contributor to the advancement of optic nerve damage [66]. Although ocular blood flow measurement with existing techniques, including OCDUS, is not yet considered a clinical tool to diagnose glaucoma, numerous studies have suggested its use in both the diagnosis and effective monitoring of glaucoma progression. Analyzing the current literature, glaucoma represents the ophthalmological disease with the most published studies (more than 100 studies) using OCDUS. In two different studies conducted respectively in 2013 and 2015, Meng et al. [67] and Xu et al. [68] concluded that patients with Primary Open-Angle Glaucoma (POAG) and Normal Pressure Glaucoma (NPG), either with or without treatment, have statistically significantly reduced PSV and EDV in the OA, CRA, and SPCAs, as well as statistically significantly elevated RI in all vessels. Most recently, the Leuven Eye Study, which is one of the largest databases for ocular blood flow in glaucoma enrolling 546 patients, showed reduced velocities in the CRA and OA in glaucoma patients, with no differences in SPCA rates between glaucoma and control groups [69]. Regarding Primary Angle-Closure Glaucoma (PACG), there are relatively few studies that have evaluated hemodynamic parameters. In a 2012 study, increased RI in the OA in POAG patients was found in comparison to PACG patients [70]. On the other hand, Cheng et al. reported that patients with well-controlled PACG may have decreased blood flow velocities and increased RI in the CRA and temporal SPCAs, as compared with healthy subjects. Also, the degree of retrobulbar hemodynamic impairment was well correlated with the degree of glaucomatous visual field damage [71]. As mentioned before, OCDUS may be an effective monitoring tool in glaucoma patients. In the existing literature, there is a unanimous consensus about decreased velocities and increased RIs to be used as biomarkers for the progression of glaucoma. In fact, many studies conclude that ocular blood flow measurement with OCDUS can be useful to determine the severity of the damage [72] and to monitor the progression of the disease, with elevated intraocular pressure, RI in the OA and SPCAs as a reliable indicator of visual field loss [73,74]. Several studies demonstrated a reduced EDV and a consequent increased RI in CRAs and SPCAs than in OAs [75,76,77,78] if compared with normal subjects: this peculiar aspect makes glaucoma different at OCDUS from other ophthalmological or systemic diseases (Figure 12), such as, for example, age-related macular degeneration (AMD), diabetes and atherosclerosis, in which OAs, CRAs and SPCAs are all involved equally with increased RI.

In different glaucomatous subjects, no significant hemodynamic changes in orbital vessels at OCDUS, nor long-term clinical benefits, were found using different local or systemic therapies [79,80,81,82,83,84,85,86,87,88,89]. Encouraging results were also obtained after surgical trabeculectomy with increased PSV, EDV and decreased RI in CRAs and SPCAs at OCDUS [90,91,92,93,94]. The beneficial effects of acupuncture have been recently demonstrated with visual function improvement and blood flow velocities increased in CRAs at OCDUS in glaucoma [95] and in different ophthalmological diseases [96].

### 3.6. Age-Related Macular Degeneration (AMD)

**Figure 13:**
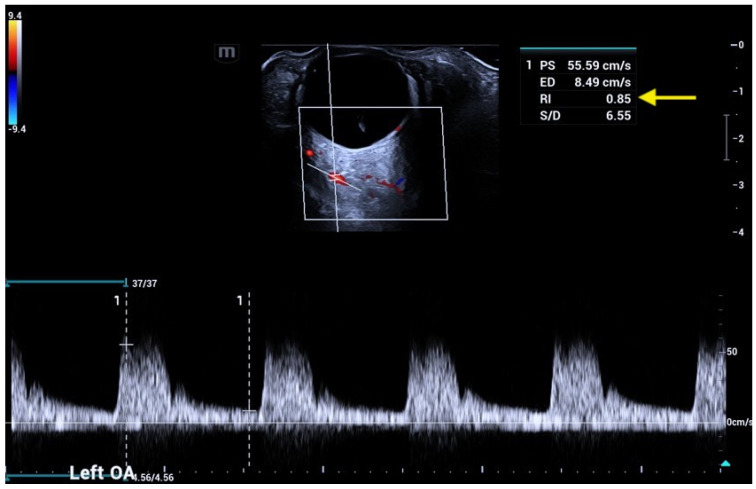
A patient affected by age-related macular degeneration (AMD) shows an increased RI of 0.85 (arrow) in the OA: usually in AMD patients, an increased RI is equally recordable at OCDUS in OAs, CRAs and SPCAs.

Age-related macular degeneration (AMD) is the leading cause of permanent and irreversible vision loss among older adults, with a higher prevalence in the Caucasian population [97]. The exact cause of the disorder remains unknown, and there are currently no means to prevent its onset or halt its progression. Evidence indicates that blood flow in the choroid of eyes affected by AMD is impaired, although the specific nature and cause of this impairment have yet to be determined [98,99]. AMD can be classified into two distinct forms: dry (non-exudative or atrophic) and wet (exudative or neovascular) AMD. All cases of AMD begin as the dry form, which affects about 85% of individuals with AMD [100]. The wet form occurs in approximately 15% of cases. Despite its lower prevalence, the wet form is responsible for 80–90% of severe vision loss in individuals with AMD [100]. While structural changes in the ocular blood vessels associated with AMD have been documented in detail, comparatively little is known about the accompanying circulatory changes. In a study by Friedman et al. [101], the combination of increased pulsatility and decreased velocity in the SPCAs observed in AMD patients was interpreted as indicative of increased vascular resistance. They suggested that the clinical manifestations of AMD may relate to the degradation of the metabolic transport function of the retinal pigment epithelium, resulting from impaired choroidal perfusion. Their study aimed to evaluate changes in ocular circulation associated with AMD. They measured ocular blood flow velocities and vessel pulsatility in volunteers with and without AMD using a color Doppler imaging unit. Spectral analyses were conducted on the OA, CRA and CRV, the temporal and nasal SPCAs, and the four vortex veins. Their results, adjusted for age, showed that pulsatility indices were higher in subjects with AMD across all arteries (CRA [*p* = 0.02]; temporal and nasal SPCAs [*p* = 0.06 and 0.002, respectively]; and OA [*p* = 0.24]). Additionally, end-diastolic blood flow velocity in the SPCAs tended to decrease in the presence of AMD. Rodrigo et al. [102] affirm that reduced choroidal perfusion linked to hyperhomocysteinemia and elevated C-reactive protein levels may contribute to the etiology of wet exudative AMD. But future research with larger patient samples is warranted to further explore these associations. Their study aims to preliminarily assess and compare plasma biomarker levels related to vascular risk in patients with and without wet exudative AMD. Additionally, it seeks to correlate these biomarkers with alterations in vascular resistance in OAs, CRAs, nasal and temporal SPCAs. The increased RI of OAs, CRAs and SPCAs, equally involved (Figure 13), was also recently confirmed in AMD at OCDUS by Finzi and colleagues [103].

Controversial and only short-term encouraging results were found using intravitreal or systemic therapies [104,105,106,107,108,109,110,111] with poor long-term beneficial effects and no significant, stable improvement of blood flow parameters at OCDUS.

### 3.7. Retinitis Pigmentosa

Retinitis pigmentosa is a genetic, severe retinal disease characterized by nyctalopia, progressive visual loss and severe impairment of the central vision in some cases: a pale, waxy optic nerve head, reduced blood flow of both choroidal and retinal vessels, and bone spicule pigment in the retina are typical [112]. A marked reduction in CRA flow velocities has usually been reported [113,114,115,116]. Reduced CRA blood flow seems to be related to impaired central visual function [117]. An encouraging improvement of retinal blood flow at OCDUS and visual function was found after electro-stimulation therapies [118].

### 3.8. Myopia

In Myopia OA, CRA, and SPCAs, an RI increase and a PSV/EDV decrease were reported at OCDUS [119,120].

### 3.9. Ocular Tumors

OCDUS was used for diagnosis and monitoring after therapy in ocular and orbital tumors to assess lesion vascularization [121,122,123,124,125,126,127] and also such as real-time fusion imaging associated with other diagnostic techniques, for example, MRI [128]. To characterize and monitor orbital tumors after systemic or local therapy, for example, gamma-knife radiosurgery, contrast-enhanced ultrasonography (CEUS) was also employed using quantitative parameters [129,130,131,132,133].

## 4. Cerebrovascular Diseases

### 4.1. Carotid Cavernous Sinus Fistulas (CCSFs)

**Figure 14:**
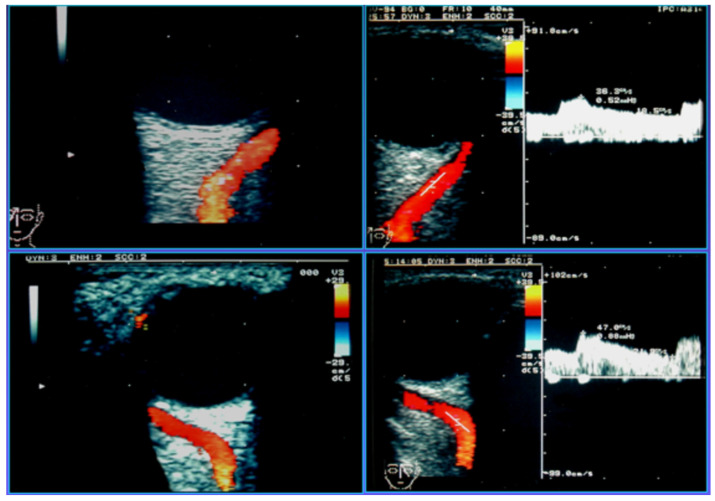
A patient affected by high-flow carotid cavernous sinus fistula (CCSF) with bilateral marked exophthalmos and bilateral SOV involvement: right and left SOV both show dilated caliber, reversed (red color), arterialized, low-resistance blood flow at OCDUS.

Carotid Cavernous Sinus Fistulas (CCSFs) represent abnormal connections between the carotid arterial system and the adjacent cavernous sinus. Based on their flow rate and source of feeder vessels, they can be classified into direct and indirect fistulas. Direct fistulas typically result from trauma and show high blood rates [134]; they consist of a direct shunt between the intracavernous portion of the internal carotid artery and the cavernous sinus. Indirect fistulas, also known as dural arteriovenous fistulas, are usually spontaneous, and their flow rates may be lower [135]. They derive from communications between the dural branches of the internal or external carotid artery and the intracranial venous sinuses. The clinical symptoms are highly dependent on the fistula’s venous drainage pattern. Usually, the arterial blood entering the cavernous sinus can exit anteriorly through the ophthalmic veins, leading to ophthalmologic symptoms due to the reversed blood flow. Congestion of the venous structures determines ocular symptoms such as exophthalmos, chemosis, conjunctival congestion, and glaucoma [135], which are more evident in high-flow fistulas, such as in direct fistulas, while dural fistulas frequently have delayed diagnosis due to more subtle manifestations [136]. Congestion of the cavernous sinus may also lead to diplopia and sometimes to intracranial hemorrhage when a cortical venous drainage is found [137]. Instead, the posterior drainage of CCSFs into the superior and inferior petrosal sinuses is usually asymptomatic [134,138]. Because the SOV reaches the cavernous sinus without a valve through the sphenoidal fissure, and no valve is present in the intracranial venous system, the SOV findings can reflect the intracranial venous hemodynamics [139]. Therefore, from 1991, based on SOV blood flow arterialization, OCDUS was used as a noninvasive diagnostic technique to diagnose and monitor CCSFs and dural fistulas. In physiological conditions, SOV usually shows a caliber of less than 1–1.5 mm at ultrasound, a flow direction moving away from the orbit depicted as blue at color, and a spectral waveform with a typical continuous venous flow with minimal differences between maximum and minimum velocities at Doppler [83]. In case of high-flow CCSF, the typical involved SOV shows a dilated caliber (>2 mm) at ultrasound, a reversed blood flow directed toward the ocular globe depicted as red at color, and a spectral waveform characterized by a pulsatile, arterialized flow with a PSV and EDV at Doppler. Usually, the arterialized blood flow is characterized by a low RI of less than 0.5, a typical signal of an arteriovenous communication [140]. OA is also characterized by a flow directed toward the globe with PSV and EDV, usually has a higher RI of more than 0.7, and a more curvilinear course than a dilated SOV with arterialized blood flow [60]. SOV’s involvement can be unilateral or bilateral (Figure 14), as the corresponding exophthalmos [60].

**Figure 15:**
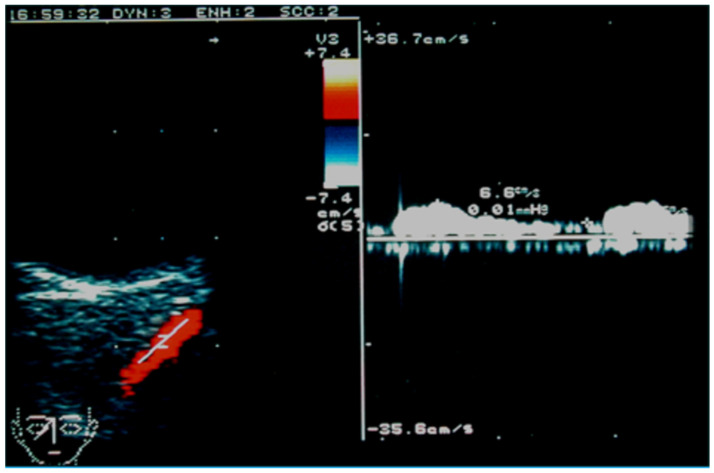
A patient affected by low-flow dural fistula with mild ocular symptoms and unilateral SOV involvement: the SOV shows a normal caliber, with reversed (red color), arterialized, low-resistance blood flow; the diagnosis may be difficult because the SOV can be undilated at OCDUS.

In case of high-flow CCSFs, also inferior ophthalmic veins, usually poorly visible at OCDUS, can also be evidenced and characterized as dilated, with reversed, arterialized, low-resistance blood flow, similarly to SOVs. In case of low-flow CCSFs or dural fistulas, the diagnosis may be more difficult because SOVs are not dilated [60] (Figure 15).

OCDUS can also be useful to demonstrate restoration of the normal course and appearance of SOV after therapeutic transcatheter embolization or gamma knife radiosurgery [141].

### 4.2. Carotid Artery Stenosis

**Figure 16:**
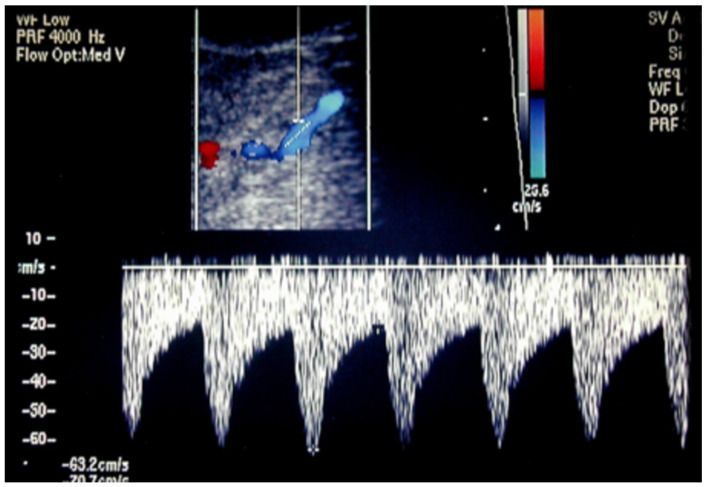
In case of need for cerebral flow redistribution due to carotid artery stenosis/occlusion, OA can appear depicted in blue with reversed blood flow at OCDUS.

Hemodynamically significant carotid artery stenosis can determine a progressive decrease in visual acuity with deep retinal hemorrhages, chronic retinal ischemic syndrome caused by chronic arterial hypoperfusion, which is frequently associated with carotid artery stenosis. Muller et al. [142] found that, out of 83 cases of vascular ocular syndromes, carotid artery stenosis of greater than 50% was more frequently observed on the side of the affected eye. Research focused on carotid artery stenosis has indicated a decrease in blood flow velocities within the CRA, the OA, or both, particularly when the stenosis exceeded 70%. In instances of occlusions, OA reversed flow was observed in 92% of cases, despite it also being able to occur in the absence of occlusions (Figure 16) in cases of need for cerebral flow redistribution [143].

Moreover, Mawn et al. noted a significant improvement in CRA velocity (from 7.4 ± 2.6 cm/s to 11.0 ± 4.6 cm/s) as well as in posterior ciliary arteries (PCAs) velocity (from 9.9 ± 4.8 cm/s to 12.7 ± 4.4 cm/s) following carotid endarterectomy [74]. This improvement of blood flow velocities of orbital arteries at OCDUS after carotid endarterectomy or angioplasty/stenting was also confirmed by other authors [144,145,146]. As mentioned above, a CRAO can be caused by embolus following a complicated carotid endarterectomy or stenting [41]. The results of these studies confirm the need for careful examination of the carotid artery in cases of low velocities found in orbital vessels. Conversely, evaluating CRA flow velocities in cases of carotid artery stenosis could be valuable for assessing the distal consequences of such stenoses and for relating chronic ischemic ocular syndrome to the presence of a severe carotid artery stenosis.

### 4.3. OCDUS as an Alternative to Transcranial Doppler to Monitor Cerebral Circulation

The noninvasive monitoring of intracranial circulation is usually performed with transcranial Doppler ultrasonography [147]. Cerebral complications after cardiac surgery are well known, with an incidence of stroke between 1 and 6% [148]. Cerebral emboli during heart surgery or other interventional cardiac or cerebral procedures can be detected with transcranial Doppler [149,150,151,152]. A limit of transcranial Doppler is the absence of the temporal acoustic window, especially in elderly women with hypotension. The CRA blood flow assessment with OCDUS during heart surgery has been investigated and compared to transcranial Doppler with corresponding findings [153]. The CRA blood flow monitoring at OCDUS during a complex cardiac surgical treatment in extracorporeal circulation has also been performed with equal values of CRA flow velocities recorded before and after treatment, suggesting the absence of significant cerebral embolisms: during the extracorporeal circulation, CRA showed a continuous flow similar to CRV with immediate restoration of a normal arterial flow at the end of the extracorporeal circulation [154].

## 5. Systemic Diseases

### 5.1. Diabetes

Type 2 diabetes is a chronic metabolic disorder characterized by elevated blood glucose levels due to impaired insulin secretion and activity. Chronic hyperglycemia leads to functional disorders of numerous organs and to their damage. Vascular lesions are typical: macroangiopathy is associated with coronary and peripheral vessels, while microangiopathic lesions can involve the eye, kidneys and nervous system [155]. One of the most serious ocular complications of diabetes is diabetic retinopathy (DR), due to progressive damage to the retinal blood vessels, which can lead to vision impairment and blindness. DR progresses through stages, from non-proliferative retinopathy, where small blood vessels weaken and leak, to proliferative retinopathy, which is associated with vitreous neovascularization, eventually leading to retinal detachment. OCDUS plays a crucial role in assessing the hemodynamic changes associated with DR. By providing quantitative data on blood flow of the orbital vessels at the retrobulbar level, OCDUS helps in the early detection of circulatory abnormalities in the eye, which is essential for timely diagnosis and management of DR [156,157]. Noureldine et al. [156] found that, dividing the studied sample into three groups (diabetic patients without DR, patients with non-proliferative DR, and patients with proliferative DR), in patients with proliferative DR, EDV of the OA was significantly lower and the RI of the OA was significantly higher, while CRA PSV and EDV were significantly lower and PCA RI was significantly higher (with a direct correlation between HbA1c). Sirkeci et al. [158] demonstrate that the development of DR could be predicted by an increased RI of OA, CRA and PCA.

DR severity was proportionally associated with CRA RI increase by several authors [159,160]. In type 1 diabetes, Pauk-Domańska et al. [160] demonstrated a statistically significant reduction in PSV and EDV of the CRA, accompanied by an increase in RI, in diabetic patients without systemic complications of the disease compared to non-diabetic subjects. In patients affected by short-term type 1 diabetes without DR successfully submitted to percutaneous intrahepatic islet transplantation, a significant increase in CRA flow velocities was found [21], while in patients affected by long-term type 1 diabetes with DR and chronic renal insufficiency successfully submitted to kidney-pancreas transplantation, no CRA flow velocity increase was recorded [161]. It is probable that CRA endothelial alterations are reversible only if treated early, and not once in the advanced stages, with DR. Controversial results were found using systemic and local therapies in diabetic patients with DR [162,163,164,165].

### 5.2. Hypertension

Despite new and effective anti-hypertensive drugs, arterial systemic hypertension is still burdened by severe morbidity and mortality, mainly related to cardiovascular and cerebrovascular diseases. Orbital vessels can be involved and, consequently, also ocular tissues. At OCDUS, hypertension-induced organ damage seems to be proportionally related to arterial RI increase [166]. For some authors, an increased RI was more evident in CRAs [14] or in SPCAs [167]. A decreased RI at OCDUS was reported using different anti-hypertensive drugs [168,169,170].

### 5.3. Takayasu Arteritis

Takayasu arteritis is an uncommon large-vessel chronic inflammatory vasculitis burdened by severe morbidity and mortality [171,172]. Takayasu arteritis usually affects the aorta and its main branches and can result in typical wall thickening, fibrosis, stenosis and thrombosis [173,174]. Diagnosis and management have been widely described [175,176]. Ophthalmological involvement with retinal vasculitis has also been reported [177,178,179]. An increased OA and CRA RI at OCDUS was recorded [180,181]. Severe cases leading to CRAO [182,183,184] and CRVO [185,186] were also reported.

### 5.4. Graves’ Disease

Graves’ disease is an autoimmune disorder where autoantibodies target the thyroid’s TSH receptor, resulting in increased synthesis and secretion of thyroid hormones. It is the most common cause of hyperthyroidism [187,188] and its prevalent extrathyroidal manifestation is Graves’ orbitopathy (GO), which occurs in up to 25% of patients, presenting with symptoms such as conjunctival redness, periorbital swelling, eyelid retraction, and proptosis. GO is influenced by a combination of inflammatory changes, external compression from enlarged extraocular muscles, increased orbital fat, elevated intraocular pressure, and systemic effects of hyperthyroidism. These factors can compromise orbital perfusion and, if left unchecked, may lead to irreversible visual loss. At OCDUS, the GO congestive phase is characterized by reduced SOV flow [189,190] not recordable in the fibrotic phase after treatment [191]. Similarly, the active congestive phase is marked by elevated OA and CRA RI, significantly reduced after orbital decompression [192,193]. Orbital decompression can improve SOV flow and decrease the RI of CRA and OA. Thus, sequential evaluation of orbital hemodynamic changes can enhance clinical scoring systems for monitoring and planning interventions in thyroid eye disease.

### 5.5. Pseudoexfoliation Syndrome

Pseudoexfoliation syndrome is an age-related systemic disease characterized by small, white deposits of fibrillary, extracellular, pseudoexfoliative material also in ocular tissue. Pseudoexfoliation syndrome can often determine an increased intraocular pressure resulting in pseudoexfoliation glaucoma with optic nerve involvement and visual alterations. Local medical treatment usually leads to poor results in terms of long-term follow-up, while encouraging results were achieved using surgical techniques, such as Argon laser, selective laser trabeculoplasty [194] or phacoemulsification surgery [195]. Non-unequivocal findings at OCDUS were reported: reduced blood flow velocities and increased RI in SPCAs and CRAs were mainly recorded [196,197,198,199].

## 6. Conclusions

In conclusion, OCDUS presents a valuable, non-invasive, real-time tool in various ophthalmological, cerebrovascular and systemic diseases: all data provided in this narrative review are summarized in Table 1.

The ability of OCDUS to provide real-time, quantitative data on retrobulbar blood flow offers significant advantages for diagnosing, monitoring, and understanding various ocular conditions, although its application in ophthalmology remains underutilized. While other imaging techniques like OCT, CT and MRI are more widely used in eye care, OCDUS stands out for its capability to visualize small vessels and provide detailed hemodynamic data. However, in my opinion, the technique’s limited adoption in clinical practice is essentially due to two reasons: first, the lack of a color Doppler training, and second, the unavailability of high-level ultrasound technologies. In this comprehensive narrative review about OCDUS many cited reports are based on medium-level ultrasound technologies, often without OCDUS images or with images without angle correction which render velocity measurements unreliable. Once these limitations are overcome, OCDUS is poised to become an increasingly important tool in both clinical and research settings for improving the understanding and management of different ophthalmological, cerebrovascular or systemic diseases with ocular involvement.

## Figures and Tables

**Table 1:** Summary of main OCDUS findings in ophthalmological, cerebrovascular and systemic diseases.

Ophthalmological Diseases		
Disease	Vessel Involved	Main OCDUS Findings
Central retinal vein occlusion (CRVO)	CRV	Reduced maxV and minV
Central retinal artery occlusion (CRAO)	CRA	Reduced PSV and EDV
Non-arteritic anterior ischemic optic neuropathy (NAION)	CRA, CRV	Reduced PSV and EDV
Orbital varix	Orbital veins	Venous dilation
Glaucoma	CRA, SPCAs	Increased RI and reduced EDV
Age-related macular degeneration (AMD)	OA, CRA, SPCAs	Increased RI and reduced EDV
Retinitis pigmentosa	CRA	Reduced PSV and EDV
Myopia	OA, CRA, SPCAs	Reduced EDV and increased RI
Orbital tumors	Tumor-feeding vessels	Tumor vascularization
**Cerebrovascular Diseases**		
**Disease**	**Vessel Involved**	**Main OCDUS Findings**
Carotid cavernous sinus fistulas (CCSFs)	SOV	Dilated, reversed, arterialized low RI blood flow
Carotid artery stenosis	OA, CRA, SPCAs	Reduced PSV and EDV
Cerebral circulation monitoring	CRA	CRA flow changes reflect intracranial perfusion
**Systemic Diseases**		
**Disease**	**Vessel Involved**	**Main OCDUS Findings**
Diabetes	OA, CRA, PCAs	Reduced EDV and increased RI
Hypertension	CRA, SPCAs	Reduced EDV and increased RI
Takayasu Arteritis	OA, CRA	Reduced EDV and increased RI
Graves’ disease	SOV, OA, CRA	Reduced venous flow, reduced EDV and increased RI
Pseudoexfoliation syndrome	CRA, SPCAs	Reduced EDV and increased RI

## Data Availability

No new data were created or analyzed in this study.

## Abbreviations

The following abbreviations are used in this manuscript:

AAION	Arteritic Anterior Ischemic Optic Neuropathy
AMD	Age-Related Macular Degeneration
CCSFs	Carotid Cavernous Sinus Fistulas
CEUS	Contrast-Enhanced UltraSound
CRA	Central Retinal Artery
CRAO	Central Retinal Artery Occlusion
CRV	Central Retinal Vein
CRVO	Central Retinal Vein Occlusion
CT	Computed Tomography
CTA	Computed Tomography Angiography
DR	Diabetic Retinopathy
EDV	End Diastolic Velocity
GO	Graves’ Orbitopathy
IOV	Inferior Ophthalmic Vein
LPCAs	Long Posterior Ciliary Arteries
MRA	Magnetic Resonance Angiography
maxV	Maximum Velocity
minV	Minimum Velocity
NAION	Non-arteritic Anterior Ischemic Optic Neuropathy
NPG	Normal Pressure Glaucoma
OA	Ophthalmic Artery
OCDUS	Ocular Color Doppler UltraSound
OCT	Optical Coherence Tomography
OCT-A	OCT-Angiography
PACG	Primary Angle-Closure Glaucoma
PCAs	Posterior Ciliary Arteries
POAG	Primary Open-Angle Glaucoma
PRF	Pulse Repetition Frequency
PSV	Peak Systolic Velocity
RI	Resistive Index
RVO	Retinal Vein Occlusion
SOV	Superior Ophthalmic Vein
SPCAs	Short Posterior Ciliary Arteries
